# Kernel-Free Quadratic Surface Support Vector Regression with Non-Negative Constraints

**DOI:** 10.3390/e25071030

**Published:** 2023-07-07

**Authors:** Dong Wei, Zhixia Yang, Junyou Ye, Xue Yang

**Affiliations:** 1 College of Mathematics and Systems Science, Xinjiang University, Urumuqi 830046, China; weidongmath@163.com (D.W.); yejymath@163.com (J.Y.); yx11092021@163.com (X.Y.); 2 Institute of Mathematics and Physics, Xinjiang University, Urumuqi 830046, China

**Keywords:** regression problem, quadratic surface, kernel-free, non-negative constraints, air quality composite index dataset

## Abstract

In this paper, a kernel-free quadratic surface support vector regression with non-negative constraints (NQSSVR) is proposed for the regression problem. The task of the NQSSVR is to find a quadratic function as a regression function. By utilizing the quadratic surface kernel-free technique, the model avoids the difficulty of choosing the kernel function and corresponding parameters, and has interpretability to a certain extent. In fact, data may have a priori information that the value of the response variable will increase as the explanatory variable grows in a non-negative interval. Moreover, in order to ensure that the regression function is monotonically increasing on the non-negative interval, the non-negative constraints with respect to the regression coefficients are introduced to construct the optimization problem of NQSSVR. And the regression function obtained by NQSSVR matches this a priori information, which has been proven in the theoretical analysis. In addition, the existence and uniqueness of the solution to the primal problem and dual problem of NQSSVR, and the relationship between them are addressed. Experimental results on two artificial datasets and seven benchmark datasets validate the feasibility and effectiveness of our approach. Finally, the effectiveness of our method is verified by real examples in air quality.

## 1. Introduction

For regression problems, sometimes there is a priori information, such as the response variable increasing as the explanatory variable increases. It is more natural to expect that the the air quality will decrease when the pollution gas concentration increases. However, the model sometimes obtains regression coefficients that do not match this a priori information, which can reduce the credibility and prediction accuracy of the model. Therefore, to solve this problem, we restrict the range of values of the regression coefficients to ensure the soundness of the model.

At present, several types of constraints have been utilized, including non-negative constraints [1,2,3,4], monotonicity constraints [5,6,7,8], smoothing constraints [9,10,11], etc. Powell et al. [12] proposed a Bayesian hierarchical model for estimating constraints conditional random fields to analyze the relationship between air pollution and health. Moreover, non-negative constraints have been applied to various problems. The non-negative least squares problem (NNLS) was introduced by Lawson [13]. Chen et al. [14] presented non-negative distributed regression as an effective method specifically designed for analyzing data in wireless sensor networks. Shekkizhar et al. [15,16] proposed non-negative kernel regression to handle graph construction from data and dictionary learning. Additionally, Chapel et al. [17] proposed non-negative penalized linear regression to address the challenge of unbalanced optimal transport.

Due to its excellent generalization ability, support vector regression (SVR) [18] has been widely used in various fields, such as the financial industry [19,20] and construction industry [21,22]. However, the selection of appropriate kernel functions and their corresponding parameters can be time-consuming during experiments, prompting researchers to explore kernel-free regression models. Su proposed the non-negative constraints SVR (NNSVR) for analyzing air quality data, which is only suitable for regression problems since it cannot use kernel functions. Based on the idea of quadratic kernel-free support vector machine (QSSVC) [23], Gao et al. [24] proposed a kernel-free fuzzy reduced quadratic surface ν-support vector machine for Alzheimer’s disease classification. Zhou et al. [25] proposed a kernel-free QSSVC. For the regression problem, Ye et al. [26,27] proposed two kernel-free nonlinear regression models, quadratic surface kernel-free least squares SVR (QLSSVR) and ϵ-kernel-free soft QSSVR (ϵ-SQSSVR), respectively. Zhai et al. [28] proposed a linear twin quadratic surface support vector regression. Zheng et al. [29] developed a hybrid QSSVR and applied it to stock indices and price forecasting. These advancements have attracted significant attention from the research community seeking more efficient approaches in regression analysis without relying on kernel functions.

In this paper, a kernel-free quadratic surface support vector regression with non-negative constraints (NQSSVR) is proposed, which incorporates the idea of a kernel-free technique and non-negative constraints. The main contributions are summarized as follows.

1. NQSSVR is proposed by utilizing the kernel-free technique, which avoids the complexity of choosing the kernel functions and their parameters, and has interpretability to some extent. In fact, the task of NQSSVR is to find a quadratic regression function to fit the data, so it can achieve better generalization ability than other linear regression methods.
2. The non-negative constraints with respect to the regression coefficients are added to construct the optimization problem of NQSSVR, which can obtain a monotonically increasing regression function with explanatory variables on a non-negative interval. In some cases, the value of the response variable grows as the explanatory variable grows. For example, when exploring the air quality examples, the air quality index will increase as the concentration of gases in the air increases.
3. Both the primal and dual problems can be solved, since our method does not involve kernel functions. In the theoretical analysis, the existence and uniqueness of solutions to the primal and dual problems, as well as their interconnections, are analyzed. In addition, the properties of regression function on the domain of definition are given.
4. Numerical experiments on artificial datasets demonstrate the visualization results of the regression function obtained by our NQSSVR. The results on benchmark datasets show that the comprehensive performance of the method is relatively better than that of linear-SVR and NNSVR. In addition, more importantly, by exploring the practical application of air quality, it can be shown that our method is more applicable than QLSSVR and ϵ-SQSSVR.

The paper is structured as follows. Section 2 introduces a brief introduction to the ϵ-SQSSVR model, and some definitions and notations. In Section 3, we construct the primal and dual problems for NQSSVR and analyze the corresponding properties. Section 4 presents the results of numerical experiments conducted on datasets. Finally, Section 5 provides conclusions from this study.

## 2. Background

In this section, we give the related definitions and notations, and review the ϵ-SQSSVR model.

### 2.1. Definition and Notations

The following mathematical notations are utilized in this paper. Lowercase bold and uppercase bold represent vectors and matrices, respectively. I is the identity matrix of any size, $S^{m}$ is the set of *m*-dimensional symmetric matrices, $R^{n\times m}$ is the set of n×m dimensional matrices. Next, define the operators as follows.

**Definition** **1.** *For any symmetry matrix $U={\left(u_{kl}\right)}_{m\times m}\in S^{m}$, its half-vectorization operator can be defined as follows:*

(1) $$\mathrm{hvec}\left(U\right)={\left(u_{11},\cdots ,u_{1m},u_{22},\cdots ,u_{2m},\cdots ,u_{mm}\right)}^{T}\in R^{\frac{m(m+1)}{2}}.$$

**Definition** **2.** *For any vector $u={\left(u_{1},u_{2},\cdots ,u_{m}\right)}^{T}\in R^{m}$, define the following quadratic operator:*

(2) $$\mathrm{lvec}\left(u\right)={\left(\frac{1}{2}u_{1}u_{1},\cdots ,u_{1}u_{m},\frac{1}{2}u_{2}u_{2},\cdots ,u_{2}u_{m},\cdots ,\frac{1}{2}u_{m}u_{m}\right)}^{T}\in R^{\frac{m(m+1)}{2}}.$$

**Definition** **3.** *For a vector $u={\left(u_{1},u_{2},\cdots ,u_{m}\right)}^{T}\in R^{m}$, define the vector-to-matrix operator as follows:*

(3) $$\mathrm{mat}\left(u\right)≜\left[\begin{array}{ccccccccc} u_{1} & u_{2} & \cdots & u_{m} & 0 & \cdots & 0 & \cdots \cdots & 0\\ 0 & u_{1} & \cdots & 0 & u_{2} & \cdots & u_{m} & \cdots \cdots & 0\\ 0 & 0 & \ddots & \vdots & \vdots & \ddots & \vdots & \ddots & \vdots \\ 0 & 0 & \cdots & u_{1} & 0 & \cdots & u_{2} & \cdots \cdots & u_{m} \end{array}\right]\in R^{m\times \frac{m(m+1)}{2}}.$$

### 2.2. ϵ-SQSSVR

Given the training set

(4) $$T=\{\left(x_{1},y_{1}\right),\left(x_{2},y_{2}\right),\cdots ,\left(x_{n},y_{n}\right)\},$$

where $x_{i}\in R^{m}$, $y_{i}\in R$, i=1,2,⋯,n. The task of ϵ-SQSSVR is to seek the quadratic regression function

(5) $$g\left(x\right)=\frac{1}{2}x^{T}Wx+b^{T}x+c,$$

where $W\in S^{m}$, $b\in R^{m}$, and c∈R. To obtain the regression function (5), the optimization problem is established as follows:

(6) $$\begin{array}{ccc} \min_{W,b,c,\xi^{\left(*\right)}} & & \frac{1}{2}\sum_{i=1}^{n}{\left\|{\mathrm{Wx}}_{i}+b\right\|}^{2}+C\sum_{i=1}^{n}\left(\xi_{i}+\xi_{i}^{*}\right), \end{array}$$

(7) $$\begin{array}{ccc} s.t. & & \left(\frac{1}{2}x_{i}^{T}Wx_{i}+b^{T}x_{i}+c\right)-y_{i}⩽\epsilon +\xi_{i},\;i=1,\cdots ,n, \end{array}$$

(8) $$\begin{array}{ccc} & & y_{i}-\left(\frac{1}{2}x_{i}^{T}Wx_{i}+b^{T}x_{i}+c\right)⩽\epsilon +\xi_{i}^{*},\;i=1,\cdots ,n, \end{array}$$

(9) $$\begin{array}{ccc} & & \xi_{i}⩾0,\xi_{i}^{*}⩾0,\;i=1,2,\cdots ,n, \end{array}$$

where C>0 is a penalty parameter, and $\xi^{\left(*\right)}={\left(\xi_{1},\xi_{1}^{*},\cdots ,\xi_{n},\xi_{n}^{*}\right)}^{T}$ is the slack vector. The optimization problem (6)–(9) is a quadratic programming problem, so it can be solved directly. In addition, this model uses the quadratic surface kernel-free technique, which avoids the difficulty of choosing the kernel function and the corresponding parameters.

## 3. Kernel-Free QSSVR with Non-Negative Constraints (NQSSVR)

In this section, we establish the primal and dual problems of the kernel-free QSSVR with the non-negative constraints (NQSSVR). The properties of primal and dual problems are discussed, and the properties of the regression function with non-negative constraints are proved.

### 3.1. Primal Problem

Given the training set *T* (4), to find the regression function (5), the following optimization problem is formulated

(10) $$\begin{array}{ccc} \min_{W,b,c,\xi^{\left(*\right)}} & & \frac{1}{2}\sum_{i=1}^{n}{\left\|{\mathrm{Wx}}_{i}+b\right\|}^{2}+C\sum_{i=1}^{n}\left(\xi_{i}+\xi_{i}^{*}\right), \end{array}$$

(11) $$\begin{array}{ccc} s.t. & & \left(\frac{1}{2}x_{i}^{T}Wx_{i}+b^{T}x_{i}+c\right)-y_{i}⩽\epsilon +\xi_{i},\;i=1,2,\cdots ,n, \end{array}$$

(12) $$\begin{array}{ccc} & & y_{i}-\left(\frac{1}{2}x_{i}^{T}Wx_{i}+b^{T}x_{i}+c\right)⩽\epsilon +\xi_{i}^{*},\;i=1,2,\cdots ,n, \end{array}$$

(13) $$\begin{array}{ccc} & & w_{kl}⩾0,b_{k}⩾0,k,l=1,\cdots ,m, \end{array}$$

(14) $$\begin{array}{ccc} & & \xi_{i}⩾0,\xi_{i}^{*}⩾0,i=1\cdots ,n, \end{array}$$

where $W={\left(w_{kl}\right)}_{m\times m}\in S^{m}$, $b=\left(b_{1},\cdots ,b_{m}\right)\in R^{m}$, c∈R. $w_{kl}⩾0,b_{k}⩾0,k,l=1,\cdots ,m$ mean that each component of W and b is greater than or equal to zero. C>0 is the penalty parameter, and $\xi^{\left(*\right)}={\left(\xi_{1},\xi_{2}^{*},\cdots ,\xi_{n},\xi_{n}^{*}\right)}^{T}$ is a slack vector.

In the above optimization problem (10)–(14), we impose constraints on the regression coefficients, namely $w_{kl}⩾0,b_{k}⩾0,k,l=1,\cdots ,m$. Restricting the range of values of regression coefficients can help us to obtain regression functions that are more consistent with a priori information. In addition, the optimization problem does not involve kernel functions, which can avoid the complicated process of kernel functions, and its parameters selection further reduced computation time.

According to Definitions 1–3, the primal optimization problem (10)–(14) is simplified to the following form:

(15) $$\begin{array}{ccc} \min_{z,c,\xi^{\left(*\right)}} & & \frac{1}{2}z^{T}\mathrm{Gz}+C\sum_{i=1}^{n}\left(\xi_{i}+\xi_{i}^{*}\right), \end{array}$$

(16) $$\begin{array}{ccc} s.t. & & \left(z^{T}s_{i}+c\right)-y_{i}⩽\epsilon +\xi_{i},i=1\ldots ,n, \end{array}$$

(17) $$\begin{array}{ccc} & & y_{i}-\left(z^{T}s_{i}+c\right)⩽\epsilon +\xi_{i}^{*},i=1\ldots ,n, \end{array}$$

(18) $$\begin{array}{ccc} & & z⩾0, \end{array}$$

(19) $$\begin{array}{ccc} & & \xi_{i}⩾0,\xi_{i}^{*}⩾0,i=1\ldots ,n, \end{array}$$

where $z=\left[w,\;b\right]\in R^{\frac{m(m+3)}{2}}$, w≜hvec(W), $G=\sum_{i=1}^{n}{\left(V^{i}\right)}^{T}V^{i}$, $V_{i}≜\left[\mathrm{mat}\left(x_{i}\right),I\right]\in R^{m\times (\frac{m(m+3)}{2})}$, c∈R, $I\in S^{m}$, $s_{i}≜\mathrm{lvec}\left(x_{i}\right)$. z≥0 means that each component of z is greater than or equal to to zero. The matrix G is positive semidefinite matrix, since $G=\sum_{i=1}^{n}{\left(V^{i}\right)}^{T}V^{i}\geq 0$.

### 3.2. Dual Model

Next, the Lagrange multiplier vectors $\alpha^{\left(*\right)}={\left(\alpha_{1},\alpha_{1}^{*},\cdots ,\alpha_{n},\alpha_{n}^{*}\right)}^{T}$, $\eta^{\left(*\right)}=$${\left(\eta_{1},\eta_{1}^{*},\cdots ,\eta_{n},\eta_{n}^{*}\right)}^{T}$, and $\beta \in R^{\frac{m(m+3)}{2}}$ are introduced to the optimization problem (15)–(19). The Lagrange function can be expressed as follows:

(20) $$\begin{array}{cc} L(z,c,\xi^{\left(*\right)},\alpha^{\left(*\right)},\eta^{\left(*\right)},\beta ) & =\frac{1}{2}z^{T}\mathrm{Gz}+C\sum_{i=1}^{n}\left(\xi_{i}+\xi_{i}^{*}\right)-\beta^{T}z\\ & -\sum_{i=1}^{n}\alpha_{i}\left(y_{i}+\epsilon +\xi_{i}-\left(z^{T}s_{i}+c\right)\right)-\sum_{i=1}^{n}\eta_{i}\xi_{i}\\ & -\sum_{i=1}^{n}\alpha_{i}^{*}\left(\epsilon +\xi_{i}+\left(z^{T}s_{i}+c\right)-y_{i}\right)-\sum_{i=1}^{n}\eta_{i}^{*}\xi_{i}^{*}. \end{array}$$

The Karush–Kuhn–Tuchker (KKT) conditions for problems (15)–(19) are given as follows:

(21) $$\begin{array}{ccc} & & \nabla_{z}L=\mathrm{Gz}-\sum_{i=1}^{n}\left(\alpha_{i}-\alpha_{i}^{*}\right)s_{i}-\beta =0, \end{array}$$

(22) $$\begin{array}{ccc} & & \nabla_{c}L=\sum_{i=1}^{n}\left(\alpha_{i}-\alpha_{i}^{*}\right)=0, \end{array}$$

(23) $$\begin{array}{ccc} & & \nabla_{\xi_{i}}L=C-\alpha_{i}-\eta_{i}=0,\;i=1\ldots ,n, \end{array}$$

(24) $$\begin{array}{ccc} & & \nabla_{\xi_{i}^{*}}L=C-\alpha_{i}^{*}-\eta_{i}^{*}=0,\;i=1\ldots ,n, \end{array}$$

(25) $$\begin{array}{ccc} & & \epsilon +\xi_{i}-\left(z^{T}s_{i}+c\right)+y_{i}⩾0,\xi_{i}⩾0,\;i=1,\ldots ,n, \end{array}$$

(26) $$\begin{array}{ccc} & & \epsilon +\xi_{i}^{*}-y_{i}+\left(z^{T}s_{i}+c\right)⩾0,\xi_{i}^{*}⩾0,\;i=1,\ldots ,n, \end{array}$$

(27) $$\begin{array}{ccc} & & \alpha_{i}\left(y_{i}+\epsilon +\xi_{i}-s_{i}^{T}z-c\right)=0,\alpha_{i}⩾0,\;i=1,\ldots ,n, \end{array}$$

(28) $$\begin{array}{ccc} & & \alpha_{i}^{*}\left(-y_{i}+\epsilon +\xi_{i}+s_{i}^{T}z+c\right)=0,\alpha_{i}^{*}⩾0,\;i=1,\ldots ,n, \end{array}$$

(29) $$\begin{array}{ccc} & & \eta_{i}^{*}\xi_{i}^{*}=0,\eta_{i}\xi_{i}=0,\eta_{i}^{*}⩾0,\eta_{i}⩾0,\;i=1\ldots ,n, \end{array}$$

(30) $$\begin{array}{ccc} & & \beta z=0,\beta ⩾0. \end{array}$$

According to the Equation (21),

(31) $$\begin{array}{ccc} & & z=G^{-1}\left(\sum_{i=1}^{n}\left(\alpha_{i}-\alpha_{i}^{*}\right)s_{i}+\beta \right). \end{array}$$

By substituting (21)–(30) into (20), the dual problem of the optimization problem (15)–(19) is formulated as

$$\begin{array}{ccc} \min_{\alpha^{\left(*\right)},\beta }\; & & \frac{1}{2}{\left(\sum_{i=1}^{n}\left(\alpha_{i}-\alpha_{i}^{*}\right)s_{i}+\beta \right)}^{T}G^{-1}\left(\sum_{i=1}^{n}\left(\alpha_{i}-\alpha_{i}^{*}\right)s_{i}+\beta \right) \end{array}$$

(32) $$\begin{array}{ccc} & & +\sum_{i=1}^{n}\left(\alpha_{i}+\alpha_{i}^{*}\right)\epsilon -\sum_{i=1}^{n}\left(\alpha_{i}-\alpha_{i}^{*}\right)y_{i}, \end{array}$$

(33) $$\begin{array}{ccc} s.t. & & \sum_{i=1}^{n}\left(\alpha_{i}-\alpha_{i}^{*}\right)=0, \end{array}$$

(34) $$\begin{array}{ccc} & & 0⩽\alpha_{i},\alpha_{i}^{*}⩽C,\;i=1\ldots ,n, \end{array}$$

(35) $$\begin{array}{ccc} & & \beta_{j}⩾0,\;j=1,\ldots ,\frac{m(m+3)}{2}, \end{array}$$

where $\alpha^{\left(*\right)}={\left(\alpha_{1},\alpha_{1}^{*},\cdots ,\alpha_{n},\alpha_{n}^{*}\right)}^{T}$ and $\beta ={\left(\beta_{1},\cdots ,\beta_{\frac{m(m+3)}{2}}\right)}^{T}$ are vectors, and C>0 is the penalty parameter. Since the proposed model does not involve kernel functions, both the primal and dual problems can be solved directly. Solving the primal problem saves the cost of computing the inverse matrix. In particular, when the input dimension is large, solving the dual problem is more convenient.

### 3.3. Some Theoretical Analysis

In this subsection, the theoretical properties of the primal and dual problems, as well as the regression function after adding the non-negative constraints, are analyzed into properties.

**Theorem** **1.** *Given the training set T* (4) and C>0, if G is a positive definite matrix and $\left(z^{*},c^{*},\xi^{*},\xi^{**}\right)$ is the optimal solution to the primal problem (15)–(19), then $z^{*}$ is unique.

**Proof.**  Suppose $\left(\bar{z},\bar{c},\bar{\xi },{\bar{xi}}^{*}\right)$ and $\left(\hat{z},\hat{c},\hat{\xi },{\hat{\xi }}^{*}\right)$ are the two optimal solutions of the primal problem. There exists μ∈(0,1) such that the following equation holds:

(36) $$\left(z,c,\xi ,\xi^{*}\right)=\mu \left(\bar{z},\bar{c},\bar{\xi },{\bar{\xi }}^{*}\right)+\left(1-\mu \right)\left(\hat{z},\hat{c},\hat{\xi },{\hat{\xi }}^{*}\right).$$

Clearly, $\left(z,c,\xi ,\xi^{*}\right)$ is also an optimal solution to the optimization problem (15)–(19), so the following inequalities hold:

(37) $$\frac{1}{2}z^{T}\mathrm{Gz}+C\sum_{i=1}^{n}\left(\xi_{i}+\xi_{i}^{*}\right)⩾\frac{1}{2}{\bar{z}}^{T}G\bar{z}+C\sum_{i=1}^{n}\left(\bar{\xi_{i}}+{\bar{\xi_{i}}}^{*}\right),$$

(38) $$\frac{1}{2}z^{T}\mathrm{Gz}+C\sum_{i=1}^{n}\left(\xi_{i}+\xi_{i}^{*}\right)⩾\frac{1}{2}{\hat{z}}^{T}G\hat{z}+C\sum_{i=1}^{n}\left(\hat{\xi_{i}}+{\hat{\xi_{i}}}^{*}\right).$$

Multiplying inequality (37) by μ and inequality (38) by (1−μ), then summing them up yields

(39) $$\frac{1}{2}z^{T}\mathrm{Gz}+C\sum_{i=1}^{n}\left(\xi_{i}+\xi_{i}^{*}\right)⩾\frac{\mu }{2}{\hat{z}}^{T}G\hat{z}+\frac{1-\mu }{2}{\bar{z}}^{T}G\bar{z}+C\sum_{i=1}^{n}\left(\mu \hat{\xi_{i}}+\left(1-\mu \right)\bar{\xi_{i}}+\mu {\bar{\xi_{i}}}^{*}+\left(1-\mu \right){\hat{\xi_{i}}}^{*}\right).$$

By simplifying the Formula (39), we obtain

(40) $$\frac{1}{2}{\left(\mu \bar{z}+\left(1-\mu \right)\hat{z}\right)}^{T}G\left(\mu \bar{z}+\left(1-\mu \right)\hat{z}\right)⩾\frac{\mu }{2}{\hat{z}}^{T}G\hat{z}+\frac{1-\mu }{2}{\bar{z}}^{T}G\bar{z}.$$

Then, we have

(41) $$\mu \left(1-\mu \right){\left(\bar{z}-\hat{z}\right)}^{T}G\left(\bar{z}-\hat{z}\right)⩽0.$$

Since the matrix G is a positive definite matrix and μ∈(0,1), inequality (41) holds if and only if $\bar{z}=\hat{z}$. □

**Theorem** **2.** *For the training set T* (4) and C>0, if the matrix G is positive definite, the optimal solution $\alpha^{\left(*\right)}$=${\left(\alpha_{1},\alpha_{1}^{*},\cdots ,\alpha_{n},\alpha_{n}^{*}\right)}^{T}$ of the dual problem (32)–(35) *exists and is unique, and the optimal solution of the primal problem* (15)–(19) *can be expressed as*

(42) $$\begin{array}{c} z=G^{-1}\left(\sum_{i=1}^{n}\left(\alpha_{i}-\alpha_{i}^{*}\right)s_{i}+\beta \right) \end{array}$$

(43) $$c=y_{j}+\epsilon -\left(\sum_{i=1}^{n}\left(\alpha_{i}-\alpha_{i}^{*}\right)s_{i}+\beta \right)G^{-1}s_{j},\;for\;some\;\alpha_{j}\in \left(0,C\right),$$

*or*

(44) $$c=y_{k}-\epsilon -\left(\sum_{i=1}^{n}\left(\alpha_{i}-\alpha_{i}^{*}\right)s_{i}+\beta \right)G^{-1}s_{k},\;for\;some\;\alpha_{k}^{*}\in \left(0,C\right).$$

**Proof.**  By the (21) equation in the KKT condition, we have

(45) $$\begin{array}{c} z=G^{-1}\left(\sum_{i=1}^{n}\left(\alpha_{i}-\alpha_{i}^{*}\right)s_{i}+\beta \right). \end{array}$$

If $\alpha^{\left(*\right)}$ there exists components, $\alpha_{j}$ and $\alpha_{k}^{*}$. such that $\alpha_{j}\in \left(0,C\right)$ or $\alpha_{j}^{*}=0$, $\alpha_{k}=0$ or $\alpha_{k}^{*}\in \left(0,C\right)$, by the complementary slackness conditions, $\xi_{j}=\xi_{k}^{*}=0$, we have

(46) $$c=y_{j}+\epsilon -\left(\sum_{i=1}^{n}\left(\alpha_{i}-\alpha_{i}^{*}\right)s_{i}+\beta \right)G^{-1}s_{j},\;\alpha_{j}\in \left(0,C\right),$$

or

(47) $$c=y_{k}-\epsilon -\left(\sum_{i=1}^{n}\left(\alpha_{i}-\alpha_{i}^{*}\right)s_{i}+\beta \right)G^{-1}s_{k},\;\alpha_{k}^{*}\in \left(0,C\right).$$

□

Next, the properties of the regression function (5) after adding non-negative constraints with respect to the regression coefficients are analyzed. The domain D is defined as follows:

(48) $$D=\{x={\left({\left[x\right]}_{1},\ldots ,{\left[x\right]}_{m}\right)}^{T}|{\left[x\right]}_{1}⩾0,\cdots ,{\left[x\right]}_{m}⩾0\}.$$

**Theorem** **3.** *On the domain D (48), the regression function g(x)* (5) *is monotonically non-decreasing respect to each component of x if and only if the regression coefficients have the following restrictions:*

(49) $$\begin{array}{cc} \; & w_{kl}⩾0,,k,l=1,\cdots ,m,\\ \; & b_{k}⩾0,,k=1,\cdots ,m, \end{array}$$

*where $w_{kl}⩾0,$ and $b_{k}⩾0,k,l=1,\cdots ,m$ mean that each component of W and b is greater than or equal to zero.*

**Proof.**  The function g(x) can be written as

(50) $$g\left(x\right)=\frac{1}{2}\sum_{k,l=1}^{m}{\left[x\right]}_{k}w_{kl}{\left[x\right]}_{l}+\sum_{k=1}^{m}b_{k}{\left[x\right]}_{k}+c.$$

It only remains to justification that this holds for the *k*-th component of x, the quadratic function containing ${\left[x\right]}_{k}$ can be expressed as

(51) $$g\left({\left[x\right]}_{k}\right)=\frac{1}{2}{\left[x\right]}_{k}w_{kk}{\left[x\right]}_{k}+{\left[x\right]}_{k}\sum_{l\neq k}^{m}w_{kl}{\left[x\right]}_{l}+b_{k}{\left[x\right]}_{k}+c.$$

Taking the derivative of the above equation yields

(52) $$\frac{\partial g({\left[x\right]}_{k})}{\partial {\left[x\right]}_{k}}=\sum_{l=1}^{m}w_{kl}{\left[x\right]}_{l}+b_{k}.$$

On the domain D, the function $g({\left[x\right]}_{k})$ monotonically non-decreasing is equivalent to being non-negative at the right end of the above equation, so it is a necessary and sufficient condition to prove that the latter holds as follows:

(53) $$\begin{array}{cc} \; & w_{kl}=w_{lk}⩾0,l=1,\cdots ,m,\\ \; & b_{k}⩾0,k=1,\cdots ,m. \end{array}$$

Sufficiency is obvious, and we only need to prove necessity. Supposing $b_{k}<0$ and taking $x={\left({\left[x\right]}_{1},\ldots \ldots ,{\left[x\right]}_{m}\right)}^{T}=0$, then we obtain the following:

(54) $$\frac{\partial g({\left[x\right]}_{k})}{\partial {\left[x\right]}_{k}}=\sum_{l=1}^{m}w_{kl}{\left[x\right]}_{l}+b_{k}=b_{k}<0.$$

The above relation is contrary to the known conditions. Supposing the existence of $w_{kl}<0$ with all other components being zero. Obviously, when the ${\left[x\right]}_{l}$ is sufficiently large, the following formula holds:

(55) $$\frac{\partial g({\left[x\right]}_{k})}{\partial {\left[x\right]}_{k}}=\sum_{l=1}^{m}w_{kl}{\left[x\right]}_{l}+b_{k}=w_{kl}{\left[x\right]}_{l}+b_{k}<0.$$

This is a contradict. Similarly, it can be shown that Theorem 3 holds. □

## 4. Numerical Experiments

To verify the validity of our proposed NQSSVR model, we compare it with other methods, including linear SVR (lin-SVR), SVR with Gaussian kernel (rbf-SVR), and polynomial kernel (poly-SVR), and linear SVR with non-negative constraints (NNSVR), as well as QLSSVR and ϵ-SQSSVR. The primal and dual problems of the NQSSVR method are denoted as NQSSVR(p) and NQSSVR(d), respectively. The above experiments are tested on 2 artificial datasets, 7 UCI [30] datasets, and AQCI datasets. All numerical experiments in this section are conducted on a computer equipped with a 2.50GHz (i7-9700) CPU and 8G RAM using MatlabR2016(a).

To validate the fitting performances of various methods, the following four evaluation criteria are introduced as shown in Table 1. Without loss of generality, let $\hat{y_{i}}$ and ${\bar{y}}_{i}$ be the predicted and mean values, respectively. The penalty parameters *C* and ϵ-insensitive parameter, as well as the Gaussian kernel parameter σ, are selected from $\left\{2^{i}|i=-6,-5,\cdots ,5,6\right\}$, while the polynomial kernel parameter *p* is selected from {1,2}. All methods are selected through 5-fold cross-validation to obtain the optimal parameters.

### 4.1. Artificial Datasets

The 2 artificial datasets are conducted to validate the performance of the NQSSVR model.

**Example** **1.**
(56) $$y_{i}=x_{i}+0.3+\zeta ,\;x_{i}\in \left[0,1\right],\;\zeta \in N\left(0,0.1^{2}\right),\;i=1,\cdots ,100.$$

**Figure 1:**
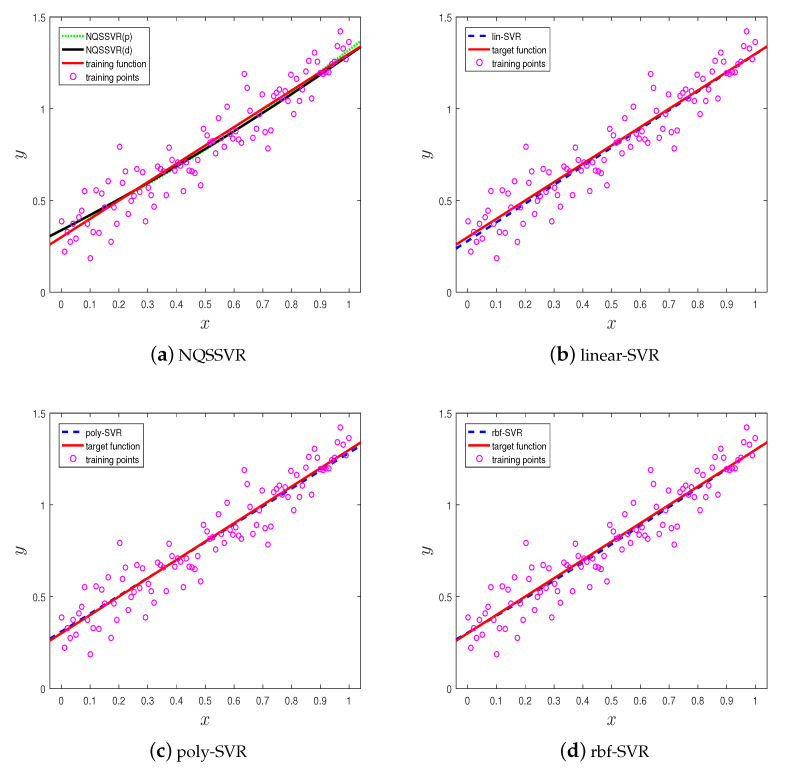
The visualization results on Example 1.

The data points are indicated by magenta “o”, the red solid line indicates the target function, and the blue dashed line indicates the regression function obtained by the kernel function method. The green dashed line and the black solid line indicate NQSSVR(p), and NQSSVR(d), respectively. The regression functions obtained by NQSSVR and different kernel functions are shown in Figure 1. From Figure 1, it can be seen that NQSSVR yields an approximately linear regression function as well as the other three methods. The regression coefficients obtained by solving the primal and dual problems of our proposed method are w=0.0001,b=1.0050,c=0.2897 and w=0.0001,b=0.9946,c=0.3097, respectively. So, NQSSVR can handle linear regression problem.

**Example** **2.**
(57) $$z_{i}=x_{i}^{2}+y_{i}^{2}+0.1+\zeta ,\;x_{i},y_{i}\in \left[0,1\right],\;\zeta \in N\left(0,0.2^{2}\right),\;i=1,\cdots ,200.$$

**Figure 2:**
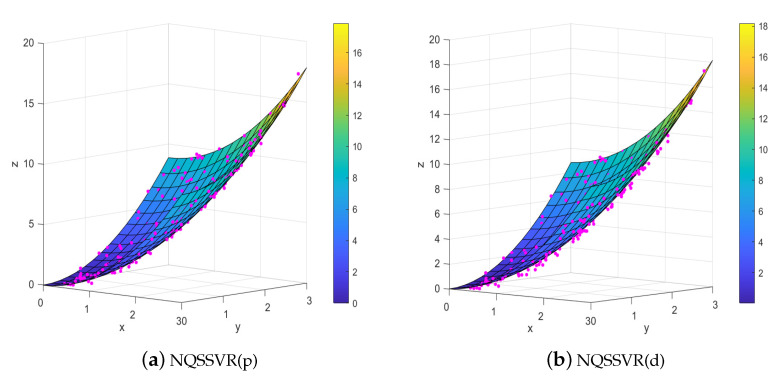
The visualization results on Example 2.

The fitting results of an artificial dataset are shown in Figure 2. The data points are denoted by “·”. Figure 2a,b present the regression surfaces obtained from the primal and dual problems, respectively. On this dataset, the regression coefficients obtained by our method are W=[0.9982,0.0000;0.0000,0.9956], $b={\left[0.0000,0.000\right]}^{T}$, c=0.1005, and W=[0.9896,0.0000;0.0000,0.9967], $b={\left[0.0000,0.0000\right]}^{T}$, c=0.1034. From Figure 2 and the regression coefficients, the quadratic surface fitted by our method can be matched to the actual distribution of the data.

Table 2 shows the results of the our proposed model and SVR with kernel functions on the two datasets mentioned above. When considering the linear regression problems, it is evident that the five models yield n similar outcomes. However, when addressing the non-linear data, our method demonstrates superior performance compared to the other three methods, as evidenced by the smaller average values of RMSE and MAE. Moreover, the difference in $R^{2}$ values between our method and the optimal result is minimal. Notably, the T2 values reveal that our method exhibits faster computation times than SVR with kernel function. This advantage comes from the fact that it does not contain a kernel function, thus eliminating the need for kernel parameter selection.

**Figure 3:**
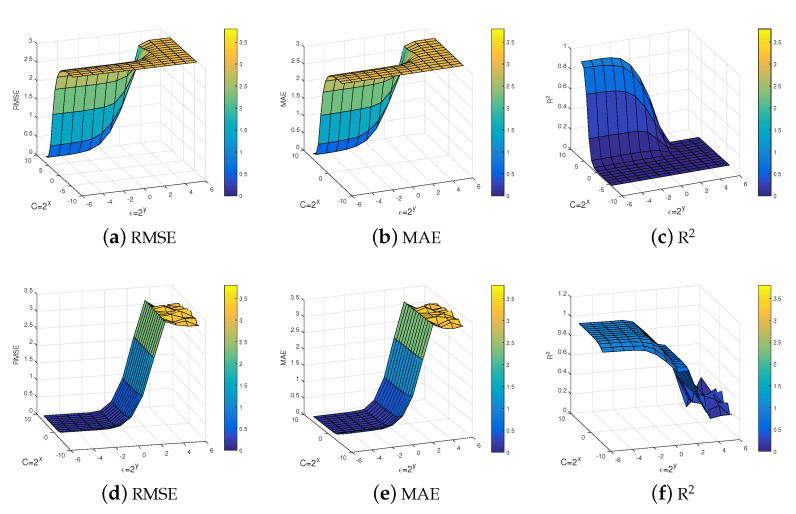
Effect of parameters on method performance NQSSVR.

For example 2, the influence of the parameter on the accuracy of our proposed method is analyzed. As can be seen by Figure 3, the penalty parameters C and insensitive loss parameter ϵ have a greater impact on the accuracy of the NQSSVR model. So, reasonable parameters can improve the accuracy of the model. In the next experiments, we choose the optimal parameters for the model within the defined parameter range.

Next, the average test time is compared for NQSSVR(p), NQSSVR(d), rbf-SVR, and poly-SVR. Since lin-SVR is only applicable to linear regression, no comparison is made here. The CPU running time of the above four methods in different dimensions and data points is shown in Table 3. Where the input dimensions m of data point are 2, 4, 8, and 16 and the number n of data points is 200, 400, 600, 800, and 1000, respectively. It is noteworthy that the time variation of NQSSVR(p) remains small as the number of data points increases for the same input dimension, outperforming both the rbf-SVR and poly-SVR methods. Moreover, when the number of data points is consistent, NQSSVR(p) exhibits shorter average test time costs compared to rbf-SVR and poly-SVR. Furthermore, as the input dimension of data points increases, the average test time for the dual problem is found to be shorter than that for the primal problem.

### 4.2. Benchmark Datasets

In this section, to further validate the reliability of the proposed method, the NQSSVR model is compared with the lin-SVR, poly-SVR, rbf-SVR, NNSVR, QLSSVR, and ϵ-SQSSVR models on seven benchmark datasets. Details of all datasets are listed in Table 4. All datasets are normalized before conducting data experiments, and are divided into training datasets, test datasets and a validation datasets in a ratio of 3:1:1. All methods are compared on evaluation criteria: MAE, RMSE, T1, T2. The top two relatively better results are highlighted in bold. All results were repeated 5 times and their mean values are calculated.

Table 5 lists the regression results of the eight methods on the seven datasets. In terms of the evaluation criteria RMSE and MAE, it can be seen that NQSSVR is significantly better than lin-SVR and NNSVR. For most of the datasets, the NQSSVR model outperforms QLSSVR and ε-SQSSVR, and is not significantly different from rbf-SVR and poly-SVR. In terms of time, our method is second only to QLSSVR and outperforms other methods.

To compare the performances of our proposed method and other six methods, the Friedman test and post hoc test are employed. Initially, the Friedman test is conducted with the null hypothesis states that all methods have the same performances. Furthermore, we can calculate the Friedman statistics for each evaluation criterion using the following formula.

(58) $$\tau_{\chi^{2}}=\frac{12N}{K(K+1)}\left(\sum_{i=1}^{K}R_{i}^{2}-\frac{K{\left(K+1\right)}^{2}}{4}\right),$$

(59) $$\tau_{F}=\frac{\left(N-1\right)\tau_{\chi^{2}}}{N\left(K-1\right)-\tau_{\chi^{2}}},$$

where *N* and *K* are, respectively, the numbers of datasets and methods, $R_{i}$ is the average rank of the *i*-th method.

According to the Formula (59), the Friedman statistics corresponding to the three criteria are 12.2124, 13.8361 and 35.1600, respectively. Next, for α = 0.05, the critical value of Friedman statistic is calculated to be $F_{\alpha }=2.2371$. Since the Friedman statistic on each regression criteria is greater than $F_{\alpha }$, so we reject the null hypothesis. That is, these 8 methods have significantly different performances on the 3 evaluation criteria. To further compare the difference of each method, we proceed with a post hoc test. Specifically, if the difference of average ranks for two methods is larger than the critical difference (CD), then their performances are considered to be significantly different. Where the CD value can be calculated by the Formula (60)

(60) $$CD=q_{\alpha }\sqrt{\frac{K(K+1)}{6N}}.$$

For α=0.05, we know $q_{\alpha }=3.0308$. Thus, we obtain CD=3.9685 by the Formula (60).

**Figure 4:**
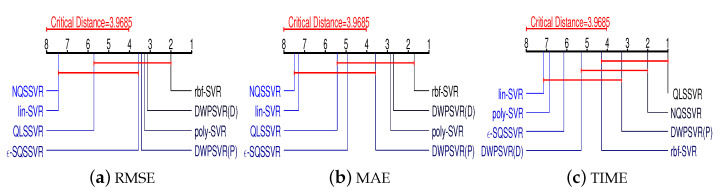
The results of Friedman test and Nemenyi post hoc test.

Figure 4 visually displays the results of Friedman test and Nemenyi post hoc test on three regression evaluation criteria, respectively. Where the average ranks of each method for three criteria are marked along an axis. The axis is turned so that the lowest (best) ranks is to the right of each criterion. Groups of methods that are not significantly different are linked by a red line. Statistically, the performance of NQSSVR (p) is not significantly different from rbf-SVR and poly-SVR in terms of RMSE, MAE. And our method ranks better than both the kernel-free quadratic surface and lin-SVR models on RMSE, MAE. In terms of time, our model ranks third and fourth, outperforming the SVR with kernel functions and ϵ-SQSSVR. In general, the comprehensive performance of our method is similar to rbf-SVR and poly-SVR, and completely superior to lin-SVR and NNSVR.

### 4.3. Air Quality Composite Index Dataset (AQCI)

This section uses two AQCI datasets, the monthly AQCI dataset and the daily AQCI dataset. These two datasets containing 18 data points and 841 data points, respectively. Each data point has six input features including nitrogen dioxide (${\mathrm{NO}}_{2}$), sulfur dioxide (${\mathrm{SO}}_{2}$), PM2.5, ozone ($O_{3}$), carbon monoxide (CO), PM10, respectively. And the output response is AQCI. Our method is compared with QLSSVR, ϵ-SQSSVR and NNSVR.

**Figure 5:**
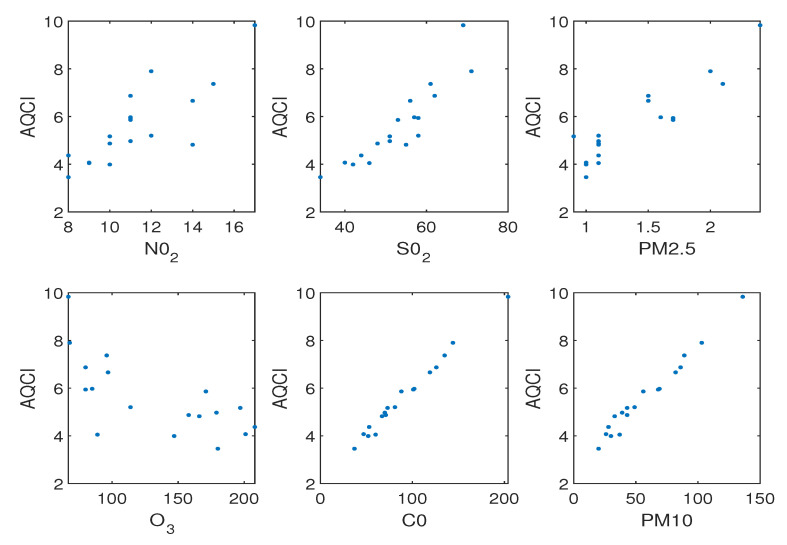
The relationship between six features and AQCI on monthly AQCI dataset.

In Figure 5, the value of AQCI has a tendency to increase as the values of the remaining five input features increase, except for the $O_{3}$ feature. Therefore, the regression function we obtain should be monotonically increasing on the monthly AQCI dataset.

Table 6 shows the experimental results of our NQSSVR and the other three methods on these two datasets. The accuracy of our model is better than that of QLSSVR and ϵ-SQSSVR on the datasets because our NQSSVR imposes non-negative constraints with respect to the regression coefficients. In addition, our model has greater accuracy than the NNSVR model. Because NNSVR can only obtain a linear regression function, but NQSSVR can obtain a quadratic regression function.

To investigate the effect of adding non-negative constraints on the accuracy of the regression function, we compare the regression coefficients W and b obtained by NQSSVR(p) with those obtained using the other three methods. Since NNSVR is a linear model, it has only linear term coefficients b and does not involve nonlinear term coefficients W. The regression coefficients obtained by the four methods are small, and for comparison purposes, we enlarge the W and b by a factor of 100 and 10, respectively, before drawing the figure. When the regression coefficient is negative, the color of the color block is closer to blue.

**Figure 6:**
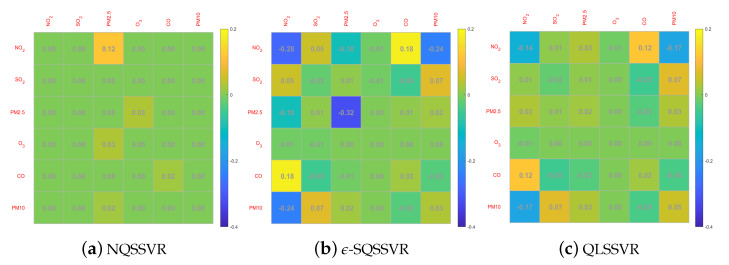
W-matrices.

**Figure 7:**
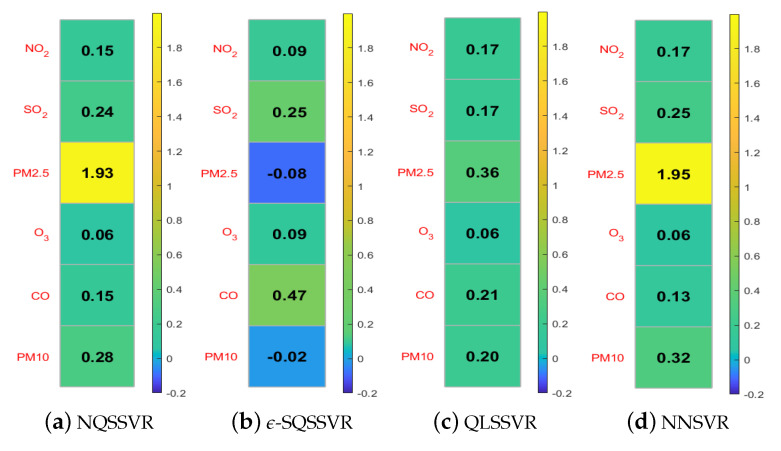
b-vectors.

We want to obtain a regression function that is monotonically increasing, by Theorem 3 it is equivalent to the non-negative constraints with respect to regression coefficients. From the Figure 6 and Figure 7, we can see that ϵ-SQSSVR and QLSSVR obtained W and b contain negative numbers, so the regression functions obtained do not match the a priori information. However, our model yields regression coefficients that all match the a priori information. Therefore, adding non-negative constraints can improve the accuracy and reasonableness of the model. Since our method can obtain a quadratic regression function, it is therefore more accurate than the linear regression function obtained by NNSVR.

**Figure 8:**
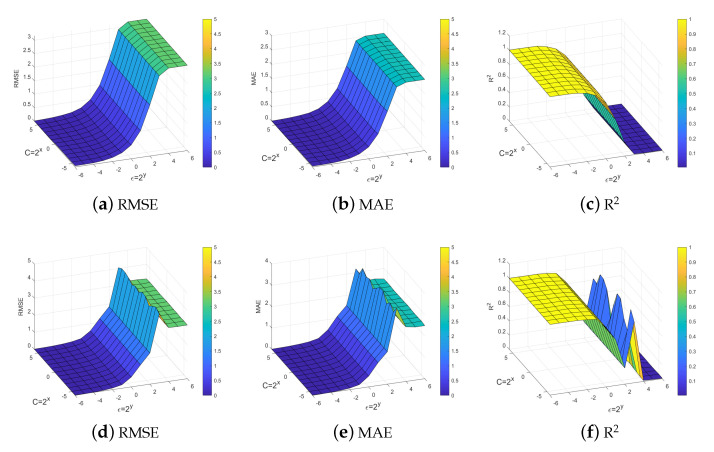
Effect of parameters on method performance NQSSVR.

Figure 8 show the effect of the parameters on the performance of our model. Here we have chosen three evaluation criteria, including RMSE, MAE, and $R^{2}$. Figure 8a–c displays the experimental results obtained for the dual problem, while Figure 8d–f presents the experimental results for the primal problem. It can be observed that the effect of the parameters on our proposed model remains significant. It is worth noting that our model receives ϵ-insensitive parameter influence more and is not very sensitive to the change of penalty parameter *C*.

## 5. Conclusions

For the regression problem, a novel kernel-free quadratic surface support vector regression with non-negative constraints (NQSSVR) is proposed by utilizing the kernel-free technique and introducing the non-negative constraints with respect to regression coefficients. Specifically, by using a quadratic surface to fit the data, the regression function is nonlinear and does not involve kernel functions, so the model is unnecessary to select kernel functions and corresponding parameters, and the obtained regression function has better interpretability. Moreover, adding non-negative constraints with respect to regression coefficients to the model ensures that the obtained regression function conforms to the monotonic non-decreasing characteristics on non-negative interval. In fact, when exploring air quality examples, there is a prior information that air quality indicators will increase with the increase in all gas concentrations in the atmosphere. Fortunately, we have proven that the quadratic regression function obtained by NQSSVR is monotonically non-decreasing on the non-negative interval if and only if the non-negative constraints with respect to the regression coefficients hold true. The results of numerical experiments on the two artificial datasets, seven benchmark datasets and air quality datasets demonstrate that our method is feasible and effective.

In this paper, we impose a non-negative restriction on the regression coefficients based on prior information. In the subsequent optimization problems, we can add different restrictions to the values of the regression coefficients based on the prior information. For example, part of the regression coefficients are restricted to non-negative intervals and part of the regression coefficients are unrestricted.

## Figures and Tables

**Table 1:** Evaluation criteria.

Evaluation Criteria	Formulas
$R^{2}$	$R^{2}=\frac{SSR}{SST}=\frac{\sum_{i=1}^{n}{\left({\hat{y}}_{i}-{\bar{y}}_{i}\right)}^{2}}{\sum_{i=1}^{n}{\left(y_{i}-{\bar{y}}_{i}\right)}^{2}}$
Mean Absolute Error (MAE)	MAE = $\frac{1}{n}\sum_{i=1}^{n}|y_{i}-{\hat{y}}_{i}|$
Root Mean Squared Error (RMSE)	RMSE = $\sqrt{\frac{1}{n}\sum_{i=1}^{n}{\left(y_{i}-{\hat{y}}_{i}\right)}^{2}}$
T1	Average test time
T2	Time to select parameters

**Table 2:** Experimental results on artificial datasets.

Datasets	Algorithms	RMSE	MAE	$R^{2}$	T1	T2
	lin-SVR	0.0901 ± 0.0010	0.0716 ± 0.0011	0.9159 ± 0.0118	0.2085 ± 0.0188	31.2781
	poly-SVR	0.0894 ± 0.0015	**0.0716** ± 0.0012	0.9502 ± 0.0134	0.1412 ± 0.0096	36.9959
Example 1	rbf-SVR	**0.0865** ± 0.0010	0.0658 ± 0.0014	**0.9680** ± 0.0153	0.1554 ± 0.0055	62.9346
	NQSSVR(p)	0.0885 ± 0.0012	0.0714 ± 0.0018	0.9066 ± 0.0260	**0.0612** ± 0.0094	**16.2336**
	NQSSVR(d)	0.0889 ± 0.0007	0.0716 ± 0.0004	0.8829 ± 0.0022	0.1278 ± 0.0019	24.2926
	lin-SVR	1.4762 ± 0.0085	1.2420 ± 0.0074	0.5141 ± 0.0021	0.4143 ± 0.0204	133.6888
	poly-SVR	0.1983 ± 0.0015	0.1567 ± 0.0018	**0.9941** ± 0.0017	0.3464 ± 0.0413	130.0461
Example 2	rbf-SVR	0.2057 ± 0.0066	0.1615 ± 0.0047	0.9899 ± 0.0014	0.4600 ± 0.0282	573.8611
	NQSSVR(p)	**0.1969** ± 0.0016	**0.1544** ± 0.0014	0.9930 ± 0.0014	**0.1850** ± 0.0139	**28.3872**
	NQSSVR(d)	0.1982 ± 0.0017	0.1552 ± 0.0017	0.9896 ± 0.0012	0.2056 ± 0.0234	32.7823

**Table 3:** Experimental results on the benchmark datasets.

Data Points∖Dimensions	Methods	m = 2	m = 4	m = 8	m = 16
n = 200	poly-SVR	3.9670 ± 0.1183	3.3426 ± 0.0813	3.6770 ± 0.0596	3.8512 ± 0.0430
	rbf-SVR	1.1682 ± 0.0318	1.0756 ± 0.0575	1.0694 ± 0.0437	1.1634 ± 0.0173
	NQSSVR(p)	**0.1592** ± 0.0146	**0.2542** ± 0.0066	**0.4346** ± 0.0193	**1.0058** ± 0.0608
	NQSSVR(d)	0.7322 ± 0.0557	0.8390 ± 0.0219	0.9502 ± 0.0449	1.1458 ± 0.0404
n = 400	poly-SVR	22.1766 ± 0.6565	21.0828 ± 0.6835	20.7110 ± 0.4407	23.2322 ± 07712
	rbf-SVR	4.1252 ± 0.1725	4.2152 ± 0.1546	4.2804 ± 0.1735	4.4358 ± 0.1441
	NQSSVR	**0.3942** ± 0.0199	**0.5334** ± 0.0219	**0.9442** ± 0.0281	**3.2328** ± 0.1464
	NQSSVR(d)	3.2784 ± 0.1339	3.8628 ± 0.0954	4.2040 ± 0.3310	4.1320 ± 0.1463
n = 600	poly-SVR	64.8220 ± 1.0185	64.9902 ± 1.6431	63.5246 ± 1.3031	69.9104 ± 2.0957
	rbf-SVR	9.6822 ± 0.4247	9.5922 ± 0.4015	10.0280 ± 0.6436	10.7400 ± 0.5887
	NQSSVR	**0.6522** ± 0.0114	**0.8818** ± 0.0064	**1.4464** ± 0.0016	**4.4658** ± 0.0995
	NQSSVR(d)	8.8504 ± 0.4567	9.4208 ± 0.3310	10.9936 ± 0.4588	12.3712 ± 1.3552
n = 800	poly-SVR	157.6060 ± 5.8607	139.2794 ± 7.3092	161.7490 ± 2.2436	163.0944 ± 4.0222
	rbf-SVR	19.5872 ± 1.2377	17.9632 ± 1.2597	18.5810 ± 0.3514	19.8404 ± 1.4977
	NQSSVR	**0.9408** ± 0.0407	**1.2254** ± 0.0537	**1.9072** ± 0.0832	**6.2810** ± 0.2554
	NQSSVR(d)	16.5370 ± 0.5350	19.1804 ± 0.8508	23.1990 ± 1.2128	26.5350 ± 0.9734
n = 1000	poly-SVR	284.1451 ± 14.6313	288.7770 ± 11.1508	272.6956 ± 10.7412	141.2644 ± 8.5463
	rbf-SVR	30.3622 ± 2.0704	32.5590 ± 2.4960	29.5694 ± 2.4395	30.9340 ± 9.9135
	NQSSVR(p)	**1.4494** ± 0.1954	**1.7290** ± 0.0310	**2.2676** ± 0.0452	**5.3260** ± 0.01937
	NQSSVR(d)	24.7908 ± 1.2614	28.3166 ± 1.4892	33.8146 ± 2.2722	44.1248 ± 1.5144

**Table 4:** Details of the benchmark datasets.

Datasets	Abbreviations	Sample Points	Attributes
Concrete Slump Test	Concrete	103	7
Computer Hardware	Computer	209	9
Yacht Hydrodynamics	Yacht	308	7
Forest Fires	Forest	517	13
Energy efficiency (Heating)	Energy(H)	768	8
Energy efficiency (Cooling)	Energy(C)	768	8
Air quality	Air	1067	6

**Table 5:** Experimental results on the datasets.

Datasets	Algorithms	RMSE	MAE	T1	T2
	lin-SVR	0.0703 ± 0.0032	0.0499 ± 0.0027	0.1618 ± 0.0122	24.2926
	poly-SVR	0.0476 ± 0.0016	0.0301 ± 0.0011	0.1708 ± 0.0130	49.2486
	rbf-SVR	0.0406 ± 0.0023	0.0275 ± 0.0015	0.1544 ± 0.0116	170.5862
	NNSVR	0.0752 ± 0.0021	0.0597 ± 0.0020	**0.0572** ± 0.0057	7.6194
Concrete	QLSSVR	0.0571 ± 0.0022	0.0453 ± 0.0017	**0.0300** ± 0.0020	5.0718
	ϵ-SQSSVR	**0.0378** ± 0.0011	**0.0242** ± 0.0014	0.3366 ± 0.0184	44.0096
	NQSSVR(p)	0.0381 ± 0.0016	0.0296 ± 0.0011	0.1608 ± 0.0051	24.0741
	NQSSVR(d)	**0.0317** ± 0.0300	**0.0267** ± 0.0016	0.1432 ± 0.0020	18.5869
	lin-SVR	0.0393 ± 0.0014	0.0199 ± 0.0003	0.5438 ± 0.0303	64.1097
	poly-SVR	0.0216 ± 0.0006	0.0084 ± 0.0002	0.5014 ± 0.0272	161.7245
	rbf-SVR	0.0179 ± 0.0011	0.0093 ± 0.0005	0.4554 ± 0.0193	507.4994
	NNSVR	0.0376 ± 0.0026	0.0217 ± 0.0009	**0.0874** ± 0.0063	11.1911
Computer	QLSSVR	0.0194 ± 0.0015	0.0099 ± 0.0004	**0.0324** ± 0.0006	5.2495
	ϵ-SQSSVR	0.0139 ± 0.0007	0.0118 ± 0.0003	0.6674 ± 0.0230	74.2415
	NQSSVR(p)	**0.0119** ± 0.0017	**0.0077** ± 0.0007	0.2132 ± 0.0115	41.8217
	NQSSVR(d)	**0.0098** ± 0.0012	**0.0063** ± 0.0005	0.3018 ± 0.0131	26.7490
	lin-SVR	0.1428 ± 0.0010	0.1129 ± 0.0007	3.2864 ± 0.0893	360.9378
	poly-SVR	**0.0676** ± 0.0204	0.0559 ± 0.0105	3.4052 ± 0.0965	798.5638
	rbf-SVR	**0.0287** ± 0.0036	**0.0204** ± 0.0011	1.6488 ± 0.0835	307.3457
	NNSVR	0.1547 ± 0.0008	0.1012 ± 0.0003	**0.1876** ± 0.0182	27.4442
Yacht	QLSSVR	0.1056 ± 0.0005	0.0817 ± 0.0006	**0.0620** ± 0.0110	8.7560
	ϵ-SQSSVR	0.0711 ± 0.0009	0.0551 ± 0.0007	1.8485 ± 0.1059	56.9315
	NQSSVR(p)	0.0965 ± 0.0006	0.0741 ± 0.0002	0.5432 ± 0.0105	67.1763
	NQSSVR(d)	0.0708 ± 0.0013	**0.0533** ± 0.0010	1.8038 ± 0.0704	167.1142
	lin-SVR	0.0528 ± 0.0010	0.0196 ± 0.0001	17.3862 ± 0.7161	1561.1308
	poly-SVR	0.0486 ± 0.0014	0.0179 ± 0.0001	6.8746 ± 0.0634	1603.4728
	rbf-SVR	0.0465 ± 0.0013	**0.0170** ± 0.0000	3.2826 ± 0.1706	812.7531
	NNSVR	0.0498 ± 0.0010	0.0198 ± 0.0001	**0.2688** ± 0.0151	45.1113
Fores	QLSSVR	0.0500 ± 0.0009	0.0186 ± 0.0001	**0.1756** ± 0.0170	29.1384
	ϵ-SQSSVR	0.0484 ± 0.0011	0.0196 ± 0.0001	0.7936 ± 0.1830	696.7686
	NQSSVR(p)	**0.0475** ± 0.0031	0.0183 ± 0.0001	0.7360 ± 0.0168	120.5714
	NQSSVR(d)	**0.0470** ± 0.0024	**0.0175** ± 0.0001	9.1456 ± 0.4374	540.1738
	lin-SVR	0.0801 ± 0.0001	0.0556 ± 0.0001	15.0628 ± 0.6460	1559.3228
	poly-SVR	**0.0298** ± 0.0001	**0.0218** ± 0.0001	12.9536 ± 0.3113	3408.8491
	rbf-SVR	**0.0237** ± 0.0004	**0.0208** ± 0.0003	5.7626 ± 0.3335	10,338.7745
	NNSVR	0.0826 ± 0.0006	0.0572 ± 0.0001	**0.3374** ± 0.0115	52.2095
Energy(H)	QLSSVR	0.0704 ± 0.0003	0.0499 ± 0.0002	**0.1482** ± 0.0081	24.4898
	ϵ-SQSSVR	0.0685 ± 0.0010	0.0464 ± 0.0004	9.8748 ± 0.1596	1065.6020
	NQSSVR(p)	0.0516 ± 0.0002	0.0405 ± 0.0002	0.8624 ± 0.0274	105.5643
	NQSSVR(d)	0.0587 ± 0.0003	0.0423 ± 0.0001	16.8058 ± 0.6380	679.5034
	lin-SVR	0.0855 ± 0.0009	0.0592 ± 0.0014	21.4692 ± 0.5119	2708.5711
	poly-SVR	**0.0623** ± 0.0001	**0.0408** ± 0.0002	28.3624 ± 0.8986	6579.0492
	rbf-SVR	**0.0398** ± 0.0018	**0.0276** ± 0.0008	9.2944 ± 0.0669	13,515.7641
	NNSVR	0.0922 ± 0.0006	0.0583 ± 0.0004	**0.5539** ± 0.0259	88.6845
Energy(C)	QLSSVR	0.0820 ± 0.0004	0.0572 ± 0.0003	**0.2338** ± 0.0409	39.7269
	ϵ-SQSSVR	0.0795 ± 0.0008	0.0512 ± 0.0005	14.7644 ± 0.1254	1782.9128
	NQSSVR(p)	0.0681 ± 0.0002	0.0476 ± 0.0001	1.4326 ± 0.0303	175.3763
	NQSSVR(d)	0.0702 ± 0.0005	0.0463 ± 0.0002	10.3206 ± 0.2744	1161.6354
	lin-SVR	0.1965 ± 0.0004	0.1637 ± 0.0002	18.5452 ± 0.9660	3563.6484
	poly-SVR	**0.1197** ± 0.0018	**0.0884** ± 0.0001	32.7722 ± 0.7051	7896.3575
	rbf-SVR	**0.1246** ± 0.0003	**0.0727** ± 0.0002	12.2458 ± 0.3911	16,768.0504
	NNSVR	0.1963 ± 0.0002	0.1038 ± 0.0002	**0.4592** ± 0.0041	77.6678
Air	QLSSVR	0.1346 ± 0.0002	0.0958 ± 0.0001	**0.1078** ± 0.0077	17.8609
	ϵ-SQSSVR	0.1265 ± 0.0003	0.1033 ± 0.0003	20.6978 ± 0.1596	2066.3358
	NQSSVR(p)	0.1385 ± 0.0001	0.0936 ± 0.0002	0.6869 ± 0.0241	122.4225
	NQSSVR(d)	0.1458 ± 0.0007	0.0965 ± 0.0006	16.4676 ± 0.7046	677.5034

**Table 6:** Results on the AQCI datasets.

Datasets	Algorithms	RMSE	MAE	$R^{2}$	T1	T2
	NNSVR	0.0274 ± 0.0001	0.0244 ± 0.0012	1.0058 ± 0.0635	0.0432 ± 0.0084	7.1174
	QLSSVR	0.0783 ± 0.0011	0.0668 ± 0.0008	0.1027 ± 0.1052	**0.0094** ± 0.0015	**0.1103**
monthly	ϵ-SQSSVR	0.1202 ± 0.0055	0.0898 ± 0.0052	0.9865 ± 0.1071	0.1356 ± 0.0534	1.8532
	NQSSVR(p)	**0.0140** ± 0.0012	**0.0115** ± 0.0011	1.0130 ± 0.0457	0.1600 ± 0.0156	2.3799
	NQSSVR(d)	0.0185 ± 0.0015	0.0156 ± 0.0011	**1.0241** ± 0.0672	0.0482 ± 0.0072	0.4796
	NNSVR	0.0072 ± 0.0003	0.0060 ± 0.0001	1.0000 ± 0.0003	2.7365 ± 0.1292	37.1562
	QLSSVR	0.0071 ± 0.0001	0.0056 ± 0.0004	1.0000 ± 0.0002	**0.1714** ± 0.0188	**2.1832**
daily	ϵ-SQSSVR	0.0071 ± 0.0002	0.0057 ± 0.0001	1.0001 ± 0.0001	32.4848 ± 0.5036	405.5820
	NQSSVR(p)	**0.0062** ± 0.0001	**0.0054** ± 0.0001	**1.0002** ± 0.0001	2.9516 ± 0.0389	43.7415
	NQSSVR(d)	0.0067 ± 0.0001	0.0058 ± 0.0001	1.0000 ± 0.0001	2.3086 ± 0.2897	28.2437

## Data Availability

All of the benchmark datasets used in our numerical experiments are from the UCI Machine Learning Repository, which are available at https://archive.ics.uci.edu/ml/351index.php (the above datasets accessed on 18 August 2021).
